# Micro-environmental changes indicate potential for subclinical intestinal tissue damage in early-age-onset colorectal cancer patients

**DOI:** 10.1093/gastro/goaf015

**Published:** 2025-02-20

**Authors:** Sean G Kraus, Katherine A Johnson, Philip B Emmerich, Linda Clipson, Cheri A Pasch, Wei Zhang, Kristina A Matkowskyj, Dustin A Deming

**Affiliations:** Division of Hematology, Medical Oncology, and Palliative Care, Department of Medicine, University of Wisconsin School of Medicine and Public Health, University of Wisconsin–Madison, Madison, WI, USA; McArdle Laboratory for Cancer Research, Department of Oncology, University of Wisconsin–Madison, Madison, WI, USA; Division of Hematology, Medical Oncology, and Palliative Care, Department of Medicine, University of Wisconsin School of Medicine and Public Health, University of Wisconsin–Madison, Madison, WI, USA; McArdle Laboratory for Cancer Research, Department of Oncology, University of Wisconsin–Madison, Madison, WI, USA; Division of Hematology, Medical Oncology, and Palliative Care, Department of Medicine, University of Wisconsin School of Medicine and Public Health, University of Wisconsin–Madison, Madison, WI, USA; McArdle Laboratory for Cancer Research, Department of Oncology, University of Wisconsin–Madison, Madison, WI, USA; McArdle Laboratory for Cancer Research, Department of Oncology, University of Wisconsin–Madison, Madison, WI, USA; University of Wisconsin Carbone Cancer Center, Madison, WI, USA; University of Wisconsin Carbone Cancer Center, Madison, WI, USA; Department of Pathology and Laboratory Medicine, University of Wisconsin–Madison, Madison, WI, USA; University of Wisconsin Carbone Cancer Center, Madison, WI, USA; Department of Pathology and Laboratory Medicine, University of Wisconsin–Madison, Madison, WI, USA; William S. Middleton Veterans Administration Health System, Madison, WI, USA; Division of Hematology, Medical Oncology, and Palliative Care, Department of Medicine, University of Wisconsin School of Medicine and Public Health, University of Wisconsin–Madison, Madison, WI, USA; McArdle Laboratory for Cancer Research, Department of Oncology, University of Wisconsin–Madison, Madison, WI, USA; University of Wisconsin Carbone Cancer Center, Madison, WI, USA

**Keywords:** early-adult-onset, colorectal cancer, versican, versikine, cancer-associated fibroblasts

## Abstract

**Background:**

While improved screening rates have contributed to an overall decrease in the incidence of colorectal cancer (CRC), the incidence of early-age-onset CRC (EAO CRC; age <50 years) has increased. Here, we characterize the genetic alterations and tumor microenvironment (TME) for EAO and later-age-onset (LAO) CRCs to identify relevant biological differences that might point to etiologic factors.

**Methods:**

A cohort of EAO (*n *=* *60) and LAO (*n *=* *93) CRC patients were evaluated for mutations by using targeted DNA sequencing and for TME differences by using immunohistochemistry and immunofluorescence. The Cancer Genome Atlas (TCGA) PanCancer Atlas colorectal adenocarcinoma cohort was evaluated for transcriptional changes between EAO (*n *=* *82) and LAO (*n *=* *510) patients.

**Results:**

*KRAS* and *BRAF* mutations were less frequent in EAO CRCs. Gene-set enrichment analysis of TCGA data revealed the downregulation of immune-related pathways in EAO CRCs. Both age cohorts had similar numbers of CD8^+^ tumor-infiltrating lymphocytes (TILs), although LAO patients had more CD4^+^ TILs and Th1-polarized CD4s. While significant associations between immune subsets and versican (VCAN), versikine, and alpha-smooth muscle actin (αSMA) were found, none of these trends differed between age cohorts. EAO patients trended towards greater VCAN accumulation in adjacent normal tissue, lower rates of VCAN proteolysis, and decreased αSMA accumulation vs LAO patients.

**Conclusions:**

Overall, established EAO cancers are similar to LAO cancers in mutational profile and key TME features. High VCAN and αSMA expression in adjacent normal colon indicates a presence of factors that are associated with increased intestinal subclinical inflammation. Future mechanistic studies will be conducted to better understand the importance of these findings and related processes should be prioritized as potential etiologic factors for EAO tumorigenesis.

## Introduction

The incidence rates of colorectal cancer (CRC) have been steadily decreasing for several decades, which is thought to be attributed to increased screening and polypectomy [1, 2]. While this trend is encouraging, decreased incidence is not observed in all ages. Since 1975, a steady increase in incidence has occurred in patients who are <50 years of age (early-age onset [EAO]), with a 2% annual increase in incidence for patients aged 20–34 and an additional 0.41% annual increase for patients who are 35–49 years of age [3]. While hereditary CRCs are more common in younger patients, the increase in cancer incidence is not related to an increase in the rate of hereditary cancers [4]. As of 2021, EAO CRCs accounted for 10% of all CRCs and, by 2030, it is anticipated that they will account for ≤25% of all rectal cancers and 10%–12% of all colon cancers [5, 6]. CRC is now the leading cause of cancer-related death in men who are <50 years of age and the second leading cause for women who are <50 years old [7]. This trend has also been observed outside of the USA, with studies from Canada [8] and Australia [9] showing similar results.

The different molecular and clinical features of EAO vs later-age-onset (LAO) CRC have been reviewed and several sources have speculated on the potential causes of this disease [4–6, 10, 11]. With the rate of hereditary cancers remaining stable, several sources have pointed towards environmental factors, including diet and lifestyle factors [4, 10]. Aside from hereditary disease, clinical and molecular characteristics that differ in rates between EAO and LAO cancers include sidedness, rates of common driving alterations, and disease stage, even after accounting for screening rates [6, 12–16]. Although these studies highlight the important molecular features of EAO CRC, studies have yet to point towards a deeper understanding of the causes of the disease.

Numerous studies have identified the tumor microenvironment (TME) of CRC as a critical component driving the progression of the disease and therapeutic resistance [17–19]. Our laboratory has established that the proteolysis of the immunoregulatory proteoglycan versican (VCAN) correlates with CD8^+^ tumor-infiltrating lymphocytes (TILs) in CRCs [20]. This work also found that ∼90% of primary CRCs have stromal VCAN expression and almost 80% have high levels of VCAN in their stroma [20]. VCAN expression in healthy tissue is generally very low; however, VCAN accumulates in response to tissue damage or inflammation and can regulate immune responses [21, 22]. Specifically, VCAN reduces T-lymphocyte motility and disrupts dendritic cell function via TLR2 binding [23, 24]. Upon proteolytic cleavage via ADAMTS (A Disintegrin And Metalloproteinase with a ThromboSpondin type 1 motif) proteases, the N-terminal fragment of VCAN termed versikine (Vkine) is released, which has been associated with improved cDC1 dendritic cell (a subset of DC that has high capacity to present tumor antigens) maturation and activation and increased T-cell infiltration [20, 25, 26]. The role of VCAN in CRC tumorigenesis has not yet been well established but, given its prevalence and potential for immunoregulation, it could be a key factor.

In addition to the immune milieu of the TME, the phenotype of cancer-associated fibroblasts (CAFs) varies widely across cancers and is an integral part of tumor biology. The mesenchymal consensus molecular subtype (CMS4) of CRC is underrepresented in the EAO cohort, though whether this finding is related to a difference in the TME between age cohorts has yet to be confirmed [13]. Alpha-smooth muscle actin (αSMA) is expressed by smooth muscle cells and fibroblasts that have been activated in the process of wound healing [27]. Its expression can be correlated with fibrosis caused by chronic inflammation in diseases such as inflammatory bowel disease and it has been shown to have increased expression in VCAN-overexpressing fibroblasts [28, 29]. Particularly, increased αSMA expression in fibroblasts has long been appreciated as an early marker of tumorigenesis and has remained a common marker for CAFs as research has expanded [30, 31]. αSMA^+^ CAFs have been associated with tumor growth and disease recurrence in CRC [32, 33]. A deeper analysis of the stromal microenvironment and its relationship with immune modulators, such as VCAN, as well as T-cell subtypes might reveal important insights into key differences in the tumor biology of young patients.

Here, we evaluate key features of CRCs and adjacent normal tissues to gain further insight into potential etiologic factors for the development of EAO CRCs. This includes determination of the differences in the molecular profile, T-cell infiltration, VCAN accumulation/proteolysis, and presence of αSMA-expressing CAFs. Additionally, we evaluate the presence of VCAN- and αSMA-expressing fibroblasts as markers of subclinical inflammation in the intestine of patients who have developed cancer.

## Materials and methods

### TCGA data analysis

The Cancer Genome Atlas (TCGA) PanCancer Atlas COADREAD cohort data were accessed via cbioportal.org [34–36]. Mutational data were compared between the 75 patients who were diagnosed aged ≤49 years vs the 517 patients who were diagnosed at age ≥50 years. For RNA-based analyses, patients with mismatch repair deficiency were excluded. Cohorts were built on age at diagnosis (<51 vs 51–60, 61–70, 71–80, 81^+^, 51^+^) and the differential expression data from each pairwise comparison were downloaded for further analysis.

### Gene-set enrichment analysis

Differential gene expression between age-group cohorts in the TCGA PanCancer Atlas COADREAD cohorts was utilized [34, 35]. Gene-set enrichment analysis was run against the Hallmark Gene Sets by using GSEA software, through the Run GSEA PreRanked function [37]. Genes were ranked by significance of differential expression by using the formula −log10(*P*-value) × (sign of fold change). Heat maps were built in R version 4.3.0 by using the package ComplexHeatmap version 2.16 [38–40].

### CRC patient samples

Matched normal and CRC samples were obtained from 153 patients through the University of Wisconsin Carbone Cancer Center BioBank (IRB #2016–0934). These patients’ cancers were distributed across stages of disease (I–IV), gender, race, and other clinical characteristics. Two representative cores of tumor tissue and one core of normal colon tissue were obtained for each patient in collaboration with surgical pathologists to ensure proper tissue sampling. These samples were distributed across two tissue microarrays. Research herein conforms to the principles outlined in the Declaration of Helsinki.

### Immunohistochemistry

Unstained tissue sections were deparaffinized and rehydrated according to standard procedures. Antigen retrieval was conducted by using citrate buffer (pH 6.0) for Vkine and αSMA staining or EDTA buffer (pH 8.0) for CD8. For VCAN immunohistochemistry (IHC), enzymatic antigen retrieval was conducted with chondroitinase ABC (0.5 U/mL in 18 mM Tris, 1 mM sodium acetate) in a humidity chamber for 1 hour at 37°C prior to antibody application [41]. Slides were incubated overnight at 4°C in primary antibody. The following primary antibodies were used: VCAN (12C5; 1:100; Developmental Studies Hybridoma Bank; University of Iowa, Iowa City, IA, USA), Vkine (Cat #PA1-1748; 1:2000; Fisher Scientific; Hampton, NH, USA), CD8a (C8/144B; 1:200; Cat #14 0085; eBioscience; San Diego, CA, USA), and αSMA (D4K9N, Cat #19245; 1:250; Cell Signaling Technologies; Danvers, MA, USA). Slides were incubated with secondary antibody (MACH 2 Rabbit, RHRP520, BioCare Medical, Pacheco, CA, USA) for 30 minutes at room temperature. Betazoid DAB buffer (BioCare Medical, SKU DS900) was used to visualize staining and hematoxylin (BioCare Medical SKU CATHE) was used for nucleic acid counterstain. CD4 and some CD8 staining was performed at the UW Health Surgical Pathology laboratory on a BenchMark ULTRA automated system. Briefly, antigen retrieval was carried out for 64 minutes in buffer CC1 (Ventana Medical Systems, Tucson, AZ, USA) and primary antibodies, (1) CD4 clone SP35, #790–4423 and (2) CD8 clone SP57, #790–4460, Ventana, were incubated for 32 and 20 minutes, respectively, and both were detected by using Ventana UltraView Universal DAB Buffer (Ventana #760–500).

### Immunofluorescence

Unstained tissue sections were deparaffinized and rehydrated according to standard procedures. Antigen retrieval was conducted by using EDTA buffer (pH 9.0, No Tween) for all targets. Autofluorescence was removed by using Vector Lab’s TrueVIEW autofluorescence quenching kit (SP-8400–15). Cells were permeabilized for transcription factors by adding 0.1% Triton X100 to primary antibody diluent. The following antibodies were used to subtype CD4^+^ TILs: CD4 (N1UG0, Cat. #14–2444-82 Thermo-Fisher), FOXP3 (D2W8E, Cat. # 98377, Cell Signaling Technologies), and Tbet (D6N8B, Cat. #13282, Cell Signaling Technologies). Primary antibodies were incubated overnight at 4°C. Appropriate secondary antibodies labeled with Alexa Fluor 488 (Invitrogen, A11001) and Alexa Fluor 647 (Invitrogen, A31573) were incubated for 30 minutes at room temperature.

### Scoring and analysis of immunohistochemistry and immunofluorescence staining

Stained slides were examined and scored by using an Olympus BX40 microscope (Olympus; Waltham, MA). For VCAN and Vkine, a four-tiered relative intensity system was used based on stromal intensity (0–3+). Experimental groups were binned as “low” if VCAN or Vkine scored 0 or 1 and “high” if VCAN or Vkine scored 2 or 3. CD4^+^ and CD8^+^ T-cell infiltration was quantified by counting the number of positive cells within the malignant epithelium (T cells that were clearly within blood vessels or supportive stroma were not scored) per 40× high-powered field (HPF). Each stain was reviewed by a surgical pathologist who was blind to the analysis. For immunofluorescence (IF) quantification, cells were imaged by using a Nikon Ti-S microscope equipped with a CMOS camera (Flash4, Hmamatsu) and counted per 20× field of view (FOV). To evaluate CD8, Treg ratios and CD8^+^ TILs were also quantified per 20× FOV.

### Mismatch repair testing

Mismatch repair status was determined by using immunohistochemistry for MLH1, MSH6, MSH2, and PMS2. The following prediluted primary Abs were used: MLH1 (M1; mouse monoclonal), MSH6 (44; mouse monoclonal), MSH2 (G219-1129; mouse monoclonal), and PMS2 (EPR3947; rabbit monoclonal; all from Ventana Medical Systems). Staining was performed on a BenchMark ULTRA automated slide staining system and was detected by using an OptiView DAB IHC Detection Kit. Absence of staining for these proteins was scored by independent pathology review (K.A.M). Non-neoplastic colonocytes and lymphocytes were used as an internal positive control.

### DNA sequencing

DNA from formalin-fixed paraffin-embedded (FFPE) cores was isolated on a Maxwell 16 AS2000 or Maxwell CSC (Promega, Madison, WI, USA) by using the Maxwell DNA LEV Plus DNA Kit (Promega #AS1135) or Maxwell CSC FFPE DNA Kit (Promega #AS1350) according to the manufacturers’ instructions. Isolated DNA was sequenced by using the QiaSeq Comprehensive Cancer Panel Kit (Qiagen #333515) and sequenced on an Illumina HiSeq 2500 or NovaSeq 6000. Sequencing analysis through variant calling was performed at the UW Biotechnology Center. Sequence reads were adapter and quality trimmed by using Skewer, aligned to Homo sapiens build 1k_v37 by using BWA-MEM, and deduplicated by using Picard (http://picard.sourceforge.net) and Je [42–44]. Base quality scores were recalibrated by using Genome Analysis Toolkit (GATK) and mutations called by using Strelka v-2.8.4 without matched controls and annotated using SNPEff [45–47]. Resulting VCF files were uploaded to the public Galaxy web platform at usegalaxy.org and cross-referenced to ClinVar’s publicly available VCF (accessed 4/29/2019) for annotation of predicted clinical response [48]. Mutations were deemed pathogenic if the ClinVar database labeled them as either “Pathogenic” or “Likely Pathogenic.” Additionally, alterations in *APC*, *KRAS*, *NRAS*, *BRAF*, *PIK3CA*, *SMAD4*, *TP53*, *MLH1*, *MSH2*, *MSH6*, *PMS2*, *CTNNB1*, and *PTEN* were evaluated for potentially pathogenic mutations not yet curated in ClinVar.

### Statistical considerations

Wilcoxon rank-sum, Jonckheere–Terpstra, and chi-squared tests were conducted as appropriate and are noted in figure legends. A *P*-value of <0.05 was considered statistically significant.

## Results

### EAO and LAO CRCs have similar clinical and molecular features

**Figure 1.**
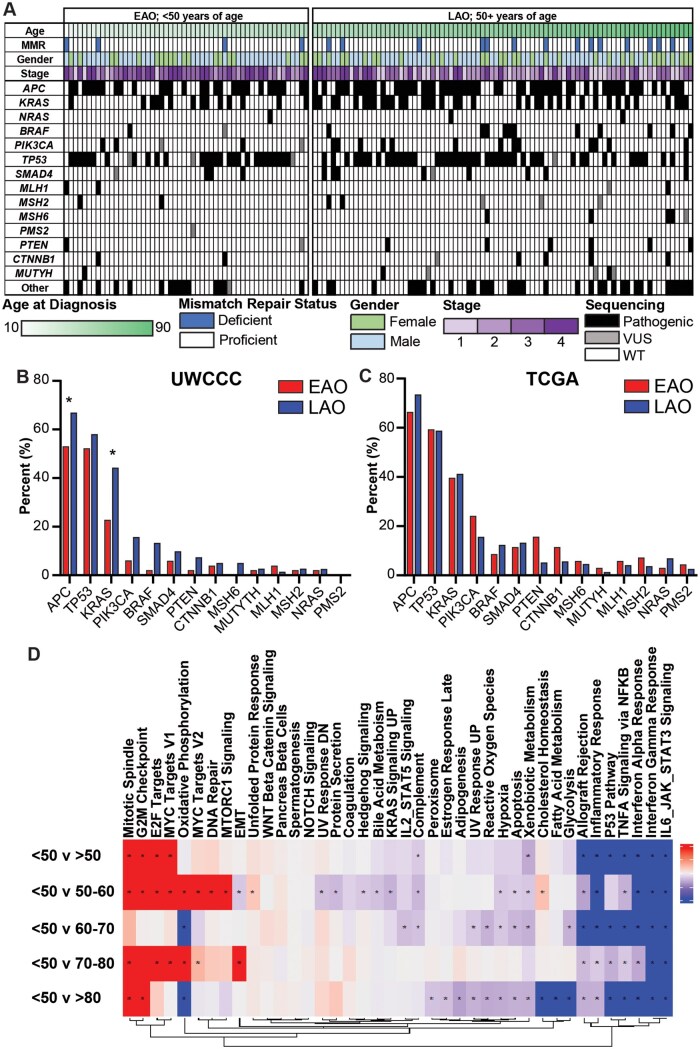
Clinical and molecular characteristics of early-age-onset (EAO) patients. Clinical and molecular analysis of the University of Wisconsin Carbone Cancer Center (UWCCC) cohort (A). Frequency of gene mutations in the UWCCC cohort (B) and The Cancer Genome Atlas (TCGA) cohorts (C). Gene-Set enrichment analysis between different age cohorts of colorectal cancer (CRC) patients (D). * *P *<* *0.05, Chi-square test, for (B), *q < 0.05 for (D).

To examine the differences between EAO and LAO CRC, we identified a cohort of 60 EAO and 93 LAO patients for which clinical data and samples were available for molecular and/or histological analysis. Molecular and clinical features for the EAO cohort and LAO cohort are presented in Figure 1A and summarized in Table 1. Between age groups, there was no significant difference in the distribution of gender or sidedness of disease. Targeted sequencing was performed to compare the molecular profiles of EAO and LAO patients. The EAO cohort in this dataset had a trend towards lower frequency in *BRAF* mutations, and a significantly lower frequency of *APC* and *KRAS* mutations (Figure 1B), in concordance with prior studies that previously identified a lower mutation frequency of *BRAF*, *KRAS*, and *APC* mutations in EAO patients [12, 13]. Additionally, the mutational frequencies observed in our samples resembled the frequencies that were found in EAO and LAO colorectal adenocarcinoma patients in the TCGA PanCancer Atlas (Figure 1C). Previous studies have found higher rates of microsatellite instability in EAO cancers [13], although there was no difference in the rate of mismatch repair deficiency between age groups in this study (*P *=* *0.35; Figure 1A and Table 1).

**Table 1.** Demographics of study cohort

Demographic	EAO (*n *=* *60)	LAO (*n *=* *93)
Mean age (range), years	36.9 (13–49)	67.4 (50–90)
Gender		
Male, *n* (%)	40 (66.7)	57 (61.3)
Female, *n* (%)	20 (33.3)	36 (38.7)
Stage		
I, *n* (%)	6 (10.0)	28 (30.1)
II, *n* (%)	10 (16.7)	21 (22.6)
III, *n* (%)	23 (38.3)	25 (26.9)
IV, *n* (%)	21 (35.0)	19 (20.4)
Disease site		
Left colon, *n* (%)	41 (68.3)	60 (64.5)
Right colon, *n* (%)	17 (28.3)	33 (35.5)
NA, *n* (%)	2 (3.3)	0 (0.0)
Mismatch repair status		
Deficient, *n* (%)	7 (11.7)	16 (17.2)
Proficient, *n* (%)	53 (88.3)	77 (82.8)

EAO = early-age-onset, LAO = late-age-onset.

### EAO CRCs display a downregulation of inflammatory pathways

To better understand the differences between EAO and LAO disease, RNA-seq data from the colorectal adenocarcinoma TCGA PanCancer Atlas dataset were analysed by using gene-set enrichment comparing patients diagnosed by age 50 years with older cohorts (Figure 1D). Demographic information about these cohorts is listed in Table 2. Consistently, the immune-related pathways, including interferon alpha and interferon gamma responses and TNFα signaling (all false discovery rate [FDR] q-value <0.001), were downregulated in the younger cohort compared with LAO patients. This indicates that these cancers may have an altered immune microenvironment warranting a deeper analysis.

**Table 2.** Demographics of The Cancer Genome Atlas cohort

Demographic	EAO (*n *=* *82)	LAO (*n *=* *510)
Mean age (range), years	45.0 (31–50)	69.5 (51–90)
Gender		
Male, *n* (%)	36 (43.9)	276 (54.1)
Female, *n* (%)	26 (56.1)	234 (45.9)
T category		
TIS, *n* (%)	1 (1.2)	0 (0.0)
T1, *n* (%)	3 (3.7)	17 (3.3)
T2, *n* (%)	10 (12.2)	93 (18.2)
T3, *n* (%)	55 (67.1)	346 (67.8)
T4, *n* (%)	13 (15.8)	54 (10.6)
Disease site		
Colon, *n* (%)	59 (71.9)	378 (74.1)
Rectum, *n* (%)	23 (28.1)	132 (25.9)

EAO = early-age-onset, LAO = late-age-onset.

### CD8 and CD4 T-cell abundance between age cohorts

**Figure 2.**
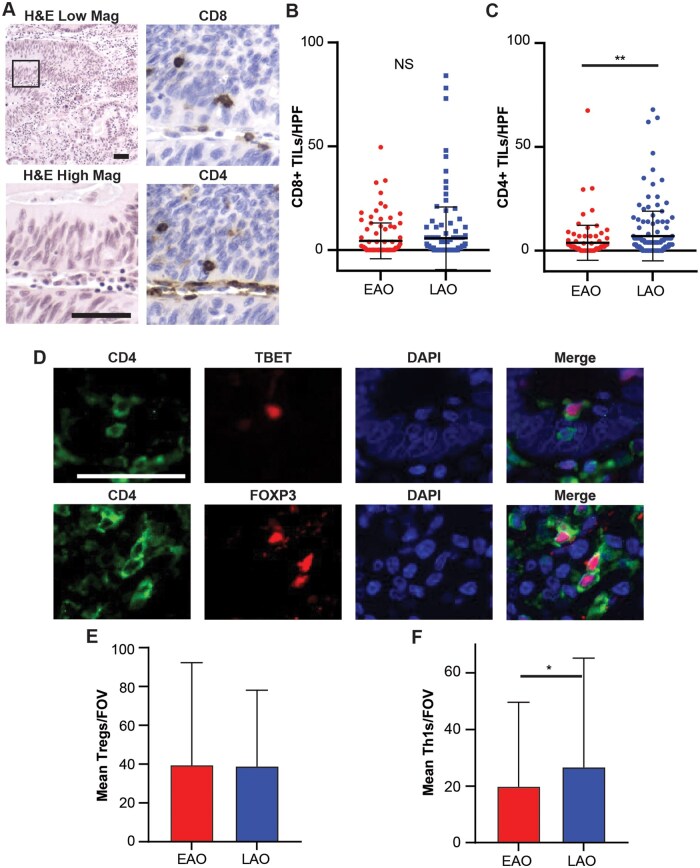
Analysis of the abundance of different T cell subsets between early-age onset (EAO) and later-age-onset (LAO) patients. (A) Example images for both CD8 and CD4 immunohistochemistry (IHC). The mean number of (B) CD8^+^ tumor-infiltrating lymphocytes (TILs) and (C) CD4^+^ TILs per high-powered field (HPF) between EAO and LAO colorectal cancer (CRC) patients. (D) Example images for CD4 subtyping. Mean number of (E) Tregs and (F) Th1s per field of view (FOV) in both EAO and LAO CRCs. *P <* *0.05, **P <* *0.01, NS not significant, Wilcoxon rank-sum test. Scale bars 50 μm.

T-cell subsets are key mediators of both antitumor and immunoregulatory cellular processes [17, 18]. To evaluate whether the altered immune signaling in younger patients seen in the TCGA cohort could be reflected by altered immune infiltration in patients, we quantified the abundance and infiltration of T-cell subsets within our study cohort. Example images for CD4 and CD8 staining are presented in Figure 2A. There was no significant difference in the mean number of CD8^+^ TILs per HPF between age cohorts (Figure 2B). Tumors from LAO patients had a mean of 7.0 CD4^+^ TILs/HPF, whereas tumors from EAO patients had a mean of 3.8 (*P *=* *0.005; Figure 2C). To further characterize the CD4^+^ TILs, immunofluorescence was performed to evaluate the presence of CD4 subtypes, Tregs (dual CD4^+^ and FOXP3^+^), and Th1s (dual CD4^+^ and TBET^+^; Figure 2D). LAO tumors had a mean of 38.9 Tregs/field of view (FOV) and EAO tumors had a mean of 39.6 Tregs/FOV (*P *>* *0.05; Figure 2E). LAO tumors had a mean of 26.7 Th1s/FOV and EAO tumors had a mean of 19.9/FOV (*P *=* *0.049, Figure 2F). Because a possible explanation for these trends could be that the distribution of disease stage at diagnosis is not the same between these two groups (Supplementary Figure S1), all T-cell populations (CD8^+^, Tregs, and Th1s) were correlated with stage to evaluate whether the stage distribution could be a confounding factor in these analyses. There was no trend observed between Tregs or CD8^+^ TILs and stage of disease. For Th1s, stage 1 cancers had a mean of 33.6 Th1s/FOV, stage 2 had a mean of 18.9 Th1s/FOV, stage 3 had a mean of 27.1 Th1s/FOV, and stage 4 cancers had a mean of 13.5 (Spearman’s correlation, *P *=* *0.034, *ρ*  =  0.14). This indicates that a higher stage disease tends to have a lower number of Th1s/FOV, potentially explaining the above observation.

### Versican accumulation within CRCs correlates with reduced T-cell abundance in both age cohorts

**Figure 3.**
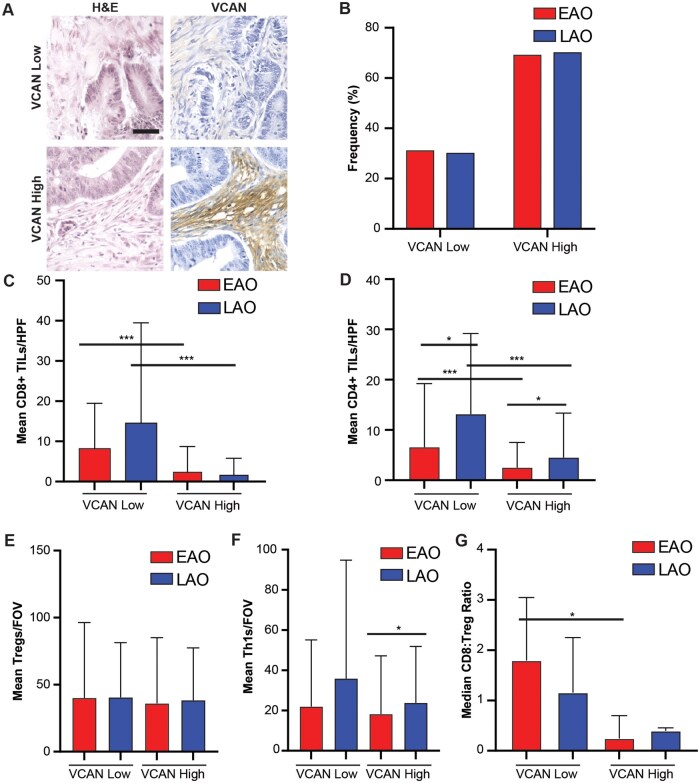
Analysis of versican (VCAN) and its association with tumor-infiltrating lymphocytes (TILs) in early-age-onset (EAO) and later-age-onset (LAO) colorectal cancer (CRC) patients. (A) Example immunohistochemistry (IHC) images for VCAN-high and VCAN-low CRCs, scale bar 50 μm. (B) The frequency of low and high VCAN accumulation in EAO and LAO CRCs. Mean number of (C) CD8^+^ TILs/high-powered field (HPF), (D) CD4^+^ TILs/HPF, (E) Tregs per field of view (FOV), (F) Th1s/FOV, and (G) median CD8:Treg ratio in VCAN-low and VCAN-high CRCs in both age cohorts. *P <* *0.05, **P <* *0.01, ***P <* *0.001, NS not significant, Wilcoxon rank-sum test.

In previous studies, we demonstrated a correlation between TIL abundance and VCAN accumulation [20]. In this analysis, we sought to evaluate whether a similar trend was observed within both EAO and LAO cancers. Examples of typical staining results are shown in Figure 3A. High levels of VCAN accumulation were common across both cohorts and staining was seen predominantly within the tumor stroma, with similar intensity distribution across the age cohorts (chi-squared, *P *=* *0.4, Figure 3B). Due to our previous findings in CRC correlating CD8^+^ TILs with VCAN accumulation, we investigated the relationship between both CD8^+^ and CD4^+^ TILs and VCAN across both age cohorts. CD8^+^ TILs were significantly increased in VCAN-low tumors (staining of 0–1+) compared with VCAN-high tumors (2–3+) for both age groups (Figure 3C). For VCAN-high tumors, LAO cancers had a mean of 1.9 CD8^+^ TILs/HPF and EAO cancer had a mean of 2.7 CD8^+^ TILs/HPF. VCAN-low tumors had a mean of 14.5 CD8^+^ TILs/HPF for LAO cancers and a mean of 8.2 TILs/HPF for EAO cancers (Figure 3C, EAO *P *=* *0.001, LAO *P *<* *0.001). Significant trends were also identified between CD4^+^ TIL accumulation and VCAN in both age cohorts (Figure 3D). In EAO patients, VCAN-low tumors had a mean of 6.6 CD4^+^ TILs/HPF and VCAN-high tumors had a mean of 2.5 (Figure 3D, *P *<* *0.001). CRCs from LAO patients had a mean of 13.2 CD4^+^ TILs/HPF in VCAN-low tumors vs a mean of 4.5 in VCAN-high (*P *<* *0.001). There was also a significant difference found between age cohorts in the mean number of CD4^+^ TILs in both VCAN-high tumors (*P *=* *0.03) and VCAN-low tumors (*P *=* *0.027). No significant trend was identified between VCAN and Treg abundance/FOV (Figure 3E). There was only a significant increase in Th1 abundance/FOV when comparing EAO VCAN-high tumors with LAO VCAN-high tumors (*P *=* *0.04; Figure 3F). Additionally, the CD8^+^ TILs-to-Treg (CD8:Treg) ratio was calculated. An increase in the CD8:Treg ratio was observed in the VCAN-low cancers, and especially so in the EAO VCAN-*- low cancers (Figure 3G).

### Versikine accumulation and its association with T-cell infiltration

With previous data demonstrating the immunostimulatory properties of Vkine, we sought to evaluate its accumulation in EAO and LAO cancers and the potential association with T-cell infiltration. Representative staining for Vkine is presented in Supplementary Figure S2A. Vkine accumulation is less common in CRC than in the adjacent normal colon [20]. Nearly 60% of cancers had high staining for Vkine (Supplementary Figure S2B). No difference was found in the accumulation of Vkine between EAO and LAO cancers (Supplementary Figure S2B). T-cell subset abundance and Vkine accumulation were evaluated across age cohorts. When assessing CD8^+^ TIL abundance in Vkine-high and Vkine-low tumors in EAO patients and LAO patients, no difference was found (Supplementary Figure S2C). When comparing the number of CD4^+^ TILs/HPF and Vkine abundance, it was found that tumors from LAO patients had a higher number of CD4^+^ TILs in Vkine-low cores, with a mean of 8.4 CD4^+^ TILs/HPF vs Vkine-high cores with a mean of 6.2 CD4^+^ TILs/HPF (Supplementary Figure S2D, *P *=* *0.005). There was also a significant difference found between Vkine-low cores across age cohorts, with Vkine-low tumors from EAO patients having a mean of 6.0 CD4^+^ TILs/HPF vs 8.4 CD4^+^ TILs/HPF in Vkine-low tumors from LAO patients (Supplementary Figure S2D, *P *=* *0.01). No significant trends were found when comparing Vkine accumulation and Treg abundance for either age cohort (Supplementary Figure S2E). There was a significant difference found for the mean number of Th1s between Vkine-high LAO cancers and Vkine-low LAO tumors (Supplementary Figure S2F). Vkine-low LAO tumors had a mean of 23.4 Th1s/FOV vs a mean of 29.3 Th1s/FOV in Vkine-high LAO tumors (*P *=* *0.047). EAO samples had no difference in the number of Th1s between Vkine-low and Vkine-high tumors.

### Versican proteolysis predicts increased T-cell abundance in both age cohorts

**Figure 4.**
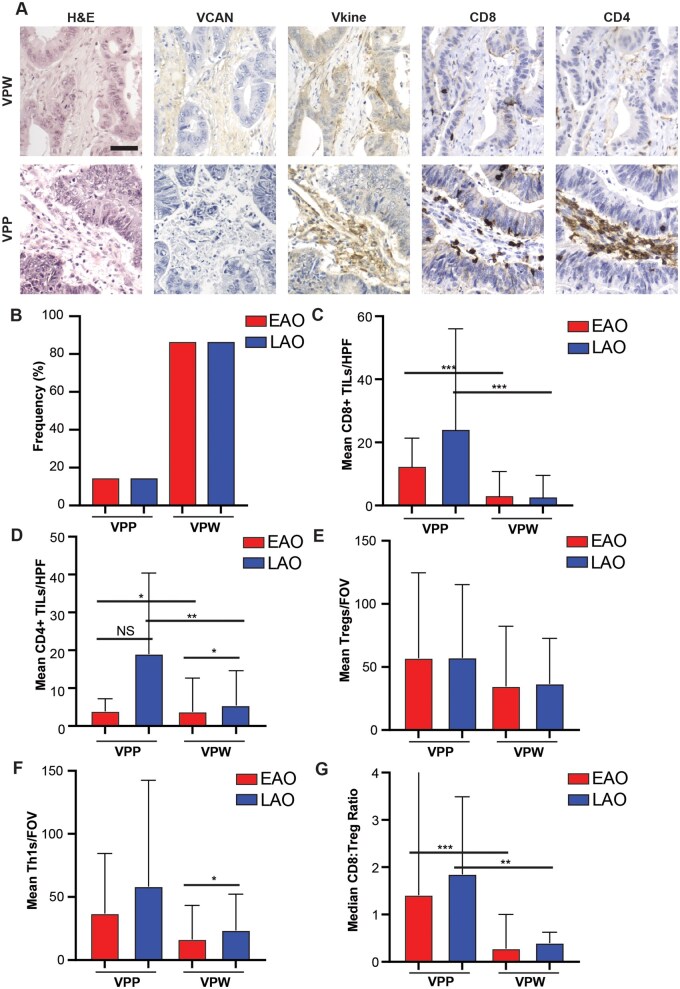
Analysis of versican (VCAN) proteolysis and its association with tumor-infiltrating lymphocytes (TILs) in early-age-onset (EAO) and later-age-onset (LAO) colorectal cancer (CRC) patients. (A) Example IHC images for VCAN proteolytic weak (VPW) and VCAN proteolytic predominant (VPP) CRCs, scale bar 50 μm. (B) Frequency of VPW and VPP CRCs in both age cohorts. Mean number of (C) CD8^+^ TILs/high-powered field (HPF), (D) CD4^+^ TILs/HPF, (E) Tregs per field of view (/FOV), (F) Th1s/FOV, and (G) median CD8:Treg ratio in VPW and VPP CRCs in both age cohorts. *P <* *0.05, **P <* *0.01, ***P <* *0.001, NS not significant, Wilcoxon rank-sum test.

As VCAN and Vkine act antagonistically in relation to their impact on T-cell infiltration, we have previously shown the importance of examining both of these biomarkers together [20]. Tissues that are VCAN-low and Vkine-high are considered VCAN proteolytic predominant (VPP) and, in prior studies, have been correlated with increased CD8^+^ T-cell infiltration [20]. All other combinations are considered VCAN proteolytic weak (VPW). We assessed the frequencies for the VPW and VPP phenotypes by using immunohistochemistry from tissues for both age cohorts (Figure 4A).

The VPP phenotype was identified in 14% of CRCs, consistently with previous results (Figure 4B) [20]. No differences in the frequencies of the VPW and VPP phenotypes were found between age cohorts. The infiltration of CD8^+^ and CD4^+^ T cells was then analysed in the context of VCAN proteolysis. In both age cohorts, VPP tumors demonstrated higher mean numbers of CD8^+^ TILs/HPF compared with VPW tumors (Figure 4C). In LAO patients, VPP tumors had a mean of 24.2 CD8^+^ TILs/HPF vs a mean of 2.8 in VPW tumors (*P *<* *0.001). In EAO patients, VPP tumors had a mean of 12.5 CD8^+^ TILs/HPF vs a mean of 3.2 in VPW tumors (Figure 4C, *P *<* *0.001). The same trends were seen when correlating CD4^+^ TIL abundance and VCAN proteolysis. LAO patients had a mean of 19.1 CD4^+^ TILs/HPF in VPP cores and a mean of 5.5 CD4^+^ TILs/HPF in VPW cores (Figure 4D, *P *=* *0.005). EAO patients had a mean of 3.9 CD4^+^ TILs/HPF in VPP cores and a mean of 3.8 CD4^+^ TILs/HPF in VPW cores (Figure 4D, *P *=* *0.05). There was also a significant difference in the number of CD4^+^ TILs/HPF found between VPW tumors from both age cohorts. VPW tumors in LAO patients had a mean of 5.5 CD4^+^ TILs/HPF, whereas VPW tumors from EAO patients had a mean of 3.8 CD4^+^ TILs/HPF (Figure 4D, *P *=* *0.02). Tregs and Th1s were assessed across the VPP and VPW phenotypes for both age cohorts (Figure 4E and 4F). A significant increase in Th1s/FOV was observed in EAO VPW cancers compared with LAO VPW cancers (*P *=* *0.012; Figure 4F). Additionally, the CD8:Treg ratio was higher in the VPP cancers for both age cohorts, with no difference between age cohorts (Figure 4G).

### Alpha-smooth muscle actin is not correlated with age of onset

**Figure 5.**
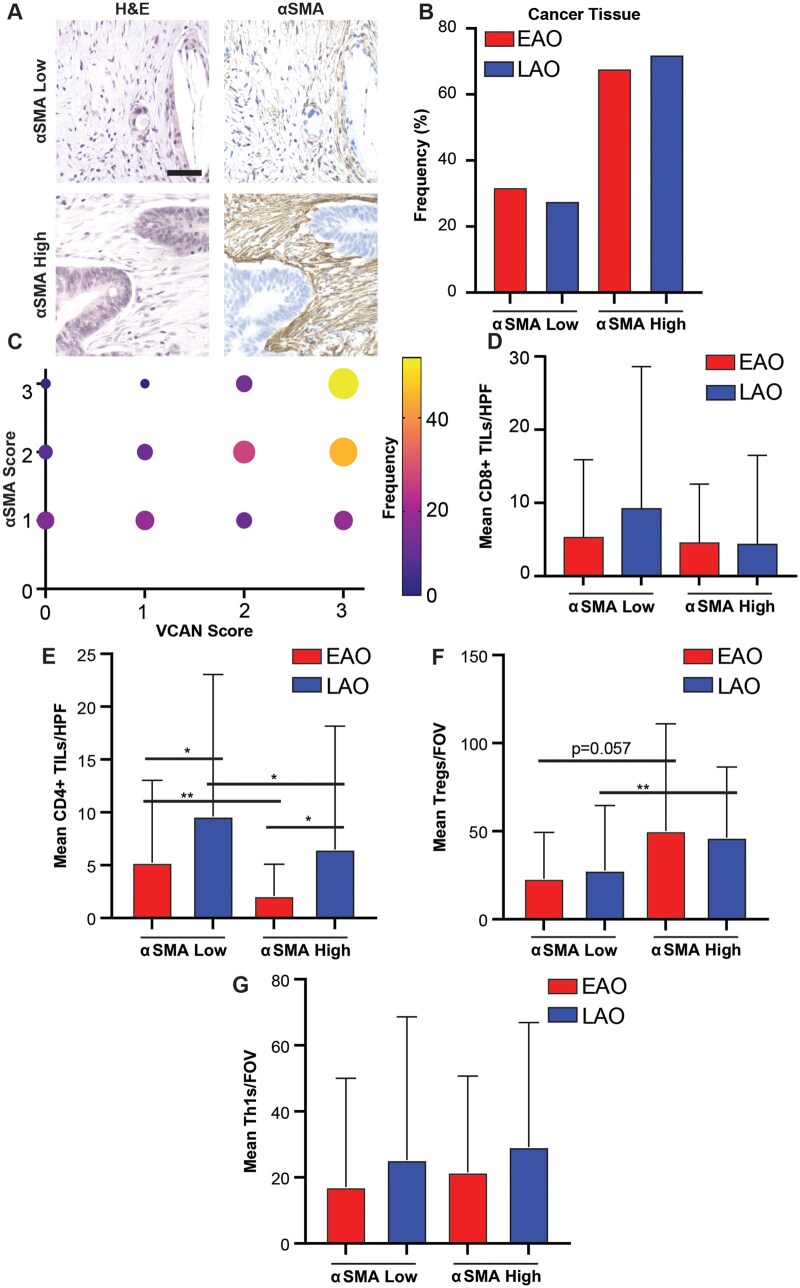
Evaluation of αSMA and its correlations with tumor-infiltrating lymphocytes (TILs) in both early-age-onset (EAO) and later-age-onset (LAO) colorectal cancer (CRC) patients. (A) Example images of αSMA IHC, scale bar 50 μm. (B) Frequency of αSMA-low and αSMA-high CRCs in both age cohorts. (C) Assessment of the co-expression of αSMA and versican (VCAN). Mean number of (D) CD8^+^ TILs/high-powered field (HPF), (E) CD4^+^ TILs/HPF, (F) Tregs per field of view (/FOV), and (G) Th1s/FOV in αSMA-low and αSMA-high CRCs in both age cohorts. *P <* *0.05, **P <* *0.01, ***P <* *0.001, NS not significant, Wilcoxon rank-sum test.

αSMA is a well-known marker of CAFs, which represents another potential avenue to evaluate the differences in the microenvironment between EAO and LAO cancers. Previous studies have found that EAO cancers have a lower rate of the mesenchymal CMS4 subtype, which can be marked by a high abundance of stroma within the tumor, but these results have not been connected back to CAFs [13]. Additionally, CAFs are known to modulate the immune microenvironment [30]. To evaluate the relationship of CAFs with onset age in CRC, we evaluated αSMA staining in the study cohorts (Figure 5A). αSMA abundance was equal between the cohorts, with αSMA-high tumors making up 68% and 72% of EAO and LAO tumor cores, respectively (Figure 5B). αSMA staining was significantly associated with the VCAN score across the entire cohort (Spearman’s *ρ*  =  0.34, *P *<* *0.001; Figure 5C), consistently with those of previous findings [28]. Despite the association with VCAN accumulation, there was no significant difference in CD8^+^ T-cell abundance between αSMA-low and αSMA-high cancers in either age cohort (Figure 5D). Similarly to VCAN, there was no significant difference in CD8^+^ T-cell infiltration between age cohorts within αSMA-low and αSMA-high cancers. For both EAO and LAO cancers, αSMA-high cancers had fewer CD4^+^ T cells/HPF than αSMA-low cancers (mean 2.1 vs 5.2 CD4^+^ TILs/HPF, *P *=* *0.003; mean 4.3 vs 9.2 CD4^+^ TILs/HPF, *P *=* *0.015, respectively; Figure 5E). Additionally, for both αSMA-low and αSMA-high cancers, EAO cancers had fewer CD4^+^ TILs/HPF than LAO cancers (Figure 5E). There was a significant difference in Treg abundance for LAO cancers, with high αSMA in tumors correlating with significantly higher Tregs than low-αSMA tumors (mean 46.4 vs 27.7 Tregs/FOV, *P *=* *0.003; Figure 5F). EAO cancers showed a similar, though not significant, trend (mean 50.2 vs 23.1 Tregs/FOV, *P *=* *0.06). There was no difference found in Th1 cells/FOV (Figure 5G). These results indicate a correlation between CAFs and T-cell abundance independently of VCAN, though not related to age of onset.

### Versican accumulation was increased and alpha-smooth muscle actin accumulation was decreased in the adjacent normal tissue of EAO CRC

**Figure 6.**
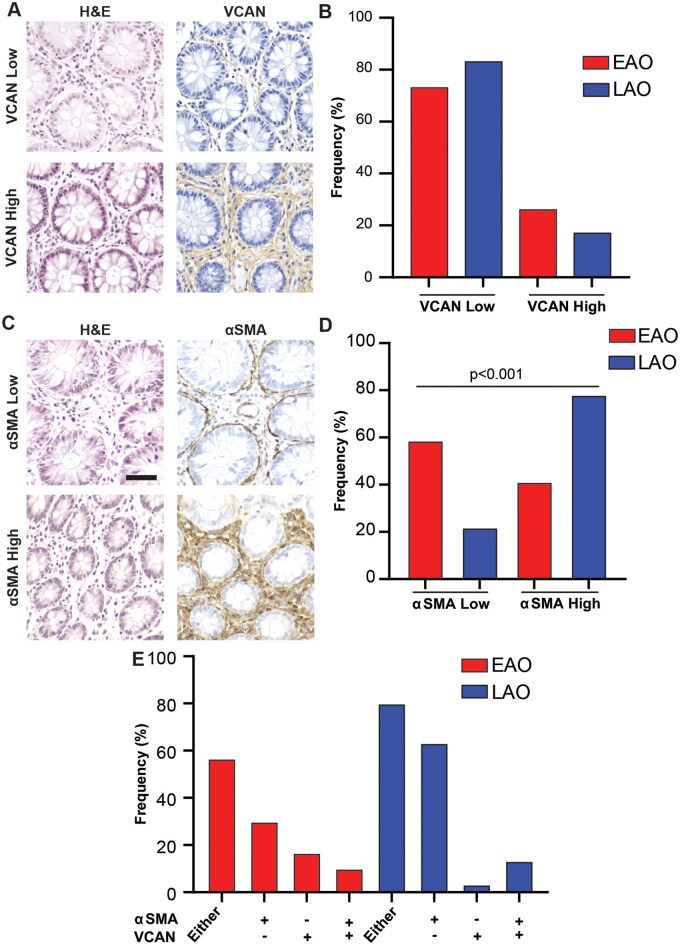
Assessment of versican (VCAN) and αSMA in adjacent normal colorectal cancer (CRC) tissues in both age cohorts. (A) Example IHC images for VCAN-high and VCAN-low adjacent normal colon. (B) Frequency of VCAN-low and VCAN-high adjacent normal colon samples in both age cohorts. (C) Example IHC images of αSMA-high and αSMA-low adjacent normal colon. (D) Frequency of αSMA-low and αSMA-high adjacent normal colon samples in both age cohorts, with significance determined by chi-square test. (E) Frequency of adjacent normal colon samples positive for αSMA, VCAN, or both in early-age-onset (EAO) and later-age-onset (LAO) samples. Scale bars 50 μm.

Previous analyses characterizing EAO CRC have focused on tumor tissue only. In our cohort, adjacent normal tissue was collected at the time of surgery and was evaluated for VCAN, Vkine, and αSMA accumulation. High levels of either VCAN or αSMA in normal tissue may indicate a predisposing level of subclinical intestinal damage that could lead to tumorigenesis. When assessing VCAN accumulation in adjacent normal tissues for both age cohorts (Figure 6A), it was found that normal tissue from EAO patients trended towards having higher rates of VCAN accumulation. In LAO adjacent normal tissues, 17% of samples were VCAN-high whereas, in adjacent normal tissue from EAO patients, 27% of samples were found to be VCAN-high (chi-squared *P *=* *0.2, Figure 6B). Marked differences were also found in αSMA abundance across normal tissues (Figure 6C). Contrary to the findings for VCAN accumulation, it was found that EAO patients had lower αSMA expression. In EAO patients, 41% of adjacent normal samples were scored as αSMA-high whereas, in LAO patients, 78% of patients were scored as αSMA-high (Figure 6D, *P *=* *0.009, chi-squared test for independence). Taking these data together, 57% of EAO samples in our cohort showed high levels of either VCAN or αSMA, indicating an altered microenvironment in this otherwise normal-appearing intestinal tissue (Figure 6E), while 80% of LAO samples fell into this category. Overall, these analyses illustrate key differences in the abundance of immunologically relevant stromal markers, indicating a difference in the exposure of inflammatory insults.

## Discussion

In this study, we characterize aspects of the molecular profiles and immune microenvironments of EAO and LAO CRCs, with the overall goal of identifying significant biological differences between these important age cohorts. The cohort studied here followed previously published trends in molecular profiles, agreeing largely with the mutational proportions of the TCGA PanCancer Atlas. Additionally, there was a significantly lower proportion of *APC* and *KRAS* mutations in the EAO cohort, as previously described in other EAO cohorts [12, 13]. These trends indicate that our study cohort is largely representative of the molecular subtypes of the population as a whole. Interestingly, while there are few differences in the clinical and molecular profiles of these cancers, our study did highlight a difference between the transcriptional profiles of EAO and LAO cancers. Enrichment analysis of TCGA data revealed that EAO cancers were downregulated in several pathways involved in immune response, including TNFα signaling and interferon alpha and gamma response. All three of these pathways are integral parts of the body’s immune system and have potential roles in the antitumor response, so decreases in the expression of these important immune pathways point towards a potential alteration in immune function in the EAO cancers [49–52].

Despite evidence in the TCGA dataset of a difference between immune functions in EAO and LAO patients, little difference was found in the infiltration of T-cell subsets between these cohorts. The abundance of clinically relevant T-cell populations was overall very similar between LAO and EAO CRC, with only a small increase in the mean number of CD4^+^ TILs and in the number of Th1s in EAO patients. The significance of this, however, is unclear. It is possible that the differences seen in Th1s/FOV and stage may partially explain the differences seen in the population of T cells by age, although further research is warranted to confirm this difference. Similar findings have been observed by Andric *et al.* [53], who found similar numbers of several relevant T-cell populations in a smaller cohort of clinically matched EAO and average-age-onset CRC patients.

We then evaluated VCAN in the TME of these cancers. VCAN is a matrix proteoglycan with immunoregulatory properties that is best known for its role in the response to tissue damage/wounds [54]. We have previously demonstrated that VCAN expression in CRCs correlates with a CD8^+^ T-cell-excluded TME [20]. Here, in this independent data set, we confirmed a similar trend. Additionally, we have previously demonstrated that Vkine, the VCAN cleavage product, has immunostimulatory properties and low levels of VCAN and high levels of Vkine (VPP phenotype) correlated with an enhanced infiltration of CD8^+^ T cells [20]. This, again, was observed in this separate cohort of patients. There was no difference in the CD8^+^ T-cell infiltration between the age cohorts. We were also able to build upon our previous knowledge of VCAN, finding that VCAN accumulation and VCAN proteolysis are also biomarkers for CD4^+^ TIL infiltration in both LAO and EAO CRC. However, when CD4^+^ T cells were subtyped and separated into age cohorts, no trend was observed between VCAN accumulation or VCAN proteolysis and either Th1s or Tregs.

VCAN expression has been related to CAFs and may be a factor of CAF abundance in the microenvironment [28]. While our study did find a significant positive correlation between the abundance of VCAN and CAF marker αSMA, the associations between these molecules and T-cell populations were markedly different. While VCAN was predictive of CD8^+^ and overall CD4^+^ T-cell infiltration, αSMA was correlated with decreased CD4^+^ T-cell infiltration as well as an increase in T regulatory cells. Despite these associations with T-cell subsets, trends remained consistent between EAO and LAO cancers, again highlighting that these cancers, once formed, are very similar in their microenvironment.

Finally, while the established tumors of LAO and EAO patients throughout our analyses were similar, there were notable indications of subclinical intestinal injury within the adjacent normal tissues of EAO patients. EAO adjacent normal tissue trended towards having greater VCAN accumulation and lower rates of VCAN proteolysis. αSMA, on the other hand, was found to have the opposite relationship to age of onset to VCAN, with lower expression of αSMA in EAO compared with LAO patients. Despite these differences in overall accumulation, the majority of EAO patients displayed either high VCAN, high αSMA, or both. αSMA is upregulated in fibroblasts during wound healing and is a well-established marker for early intestinal damage preceding tumorigenesis [27, 31]. VCAN has also been shown to have several wound-healing functions that could potentially aid tumors during the early stages of development, such as creating provisional matrices that enrich the microenvironment in both monocytes and M2 macrophages [55]. While increased markers of inflammation and injury are to be expected in the normal colon of aging individuals, it is surprising to see an abundance of these molecules in the non-neoplastic mucosa of young patients [56]. This abundance of αSMA and VCAN, indicative of injury in younger patients, warrants further investigation into potential processes that expedite subclinical intestinal damage. Future research should focus on factors that could lead to increased inflammation and subsequent injury.

These findings lay the groundwork for a new perspective in understanding the development of EAO CRC. While we found little evidence of differences between EAO and LAO cancer microenvironments, especially in immune infiltrates, our study was limited to just a few T-cell subsets. The gene-set signatures found in our study from the TCGA cohort indicated a change in immune processes that our further analysis was unable to find, indicating the potential for other immune cells to be differentially involved in these cancers. Further study into other T-cell or immune-cell subsets may yield different results and identify immunological differences between these cancers. One drawback of this study is the relatively smaller sample size of the EAO patients. As more EAO patient samples become available, we will expand our analyses to address this. In this study, the EAO cohort of patients had a higher rate of later-stage cancers relative to the LAO cohort. While this was expected due to the known correlations between EAO cancer and more advanced disease at diagnosis, some associations found herein may be confounded with associations with later-stage disease [10, 14–16]. Importantly, our studies here are purely retrospective and, from these bioinformatic and correlative studies, we cannot draw causative conclusions. These retrospective studies are helpful as a starting point to identify etiologic factors that may contribute to early tissue injury. Studying the adjacent normal tissues of individuals who have developed cancers is convenient, as these tissues are often available from surgical resections and are already known to belong to the study groups of interest. In the future, we will aim to strengthen our findings by collecting normal colon tissue from young patients with and without cancer, and associate these findings with their medical history and exposome. Additionally, prospective longitudinal studies are necessary to identify mechanisms and processes that lead to early tumorigenesis.

In this study, we evaluated clinical characteristics, mutational profiles, immunologically relevant stromal markers, and critical immune-cell populations in both LAO and EAO CRC. Overall, it was found that EAO and LAO tumors are remarkably similar in regard to the markers and cell populations evaluated in this study. However, when the adjacent normal samples were assessed for the same markers, important differences were identified between LAO and EAO samples. While we would expect the LAO normal colon to have markers of inflammation and cellular damage from years of insult, it was surprising to see high levels of αSMA and VCAN in the EAO cohort. These data could indicate that, although EAO and LAO tumors are very similar, the cause of their tumor initiation may not be. These results implicate that the increased incidence of EAO CRC may be due to a cancer-initiating inflammatory process rather than a difference that is intrinsic to the cancers themselves. Future studies should be aimed at evaluating the inflammasome of the adjacent non-neoplastic colon in patients with EAO CRC.

## Supplementary Material

goaf015_Supplementary_Data
