# Supplementary figures and images for: Micro-environmental changes indicate potential for subclinical intestinal tissue damage in early-age-onset colorectal cancer patients
Source: Gastroenterol Rep (Oxf). 2025 Feb 20;13:goaf015. doi: 10.1093/gastro/goaf015 (PMC11842056; doi:10.1093/gastro/goaf015)

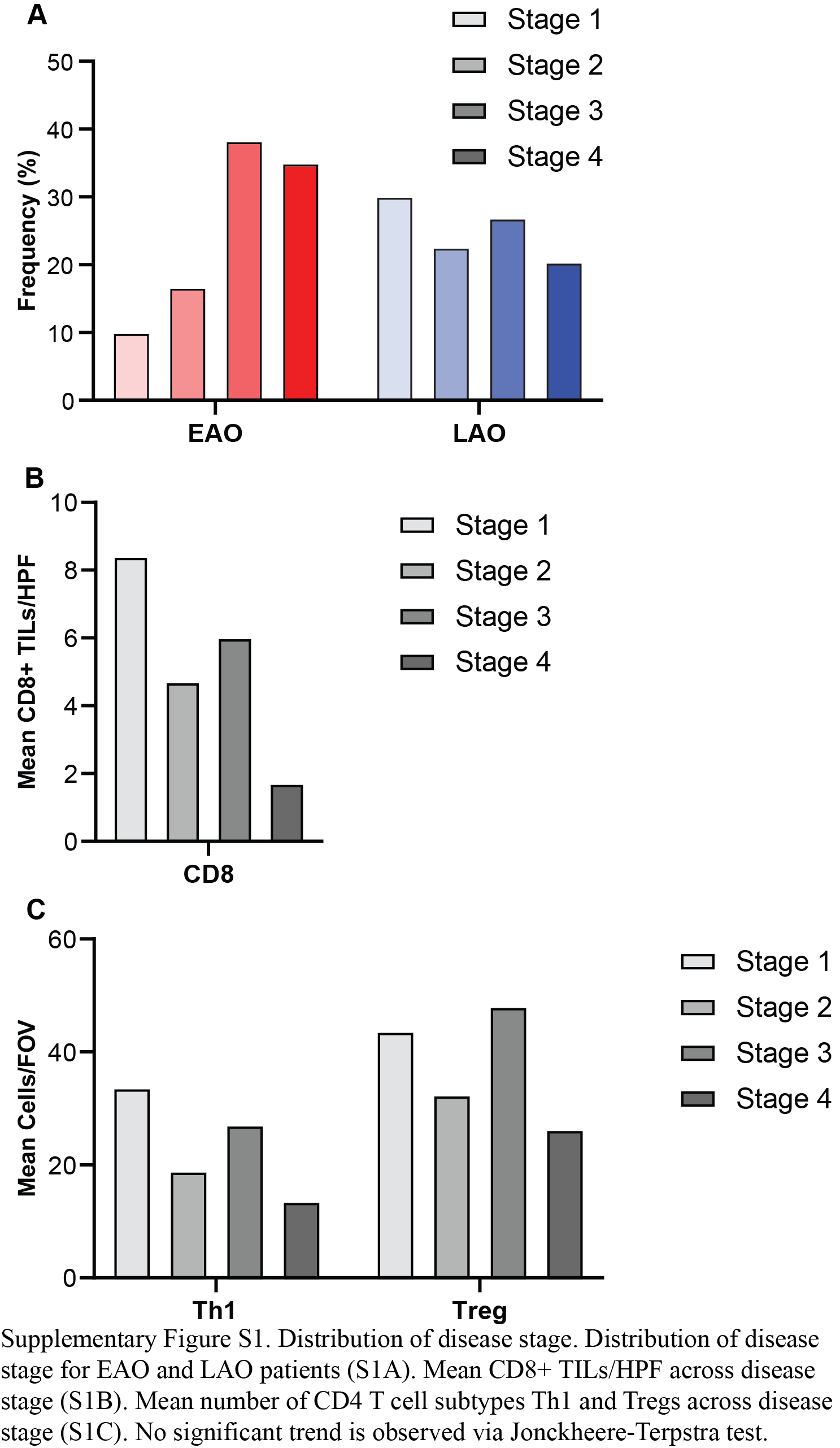

Supplement: goaf015_Supplementary_Data [file goaf015_supplementary_data.zip › Kraus_Johnson_EAO CRC Re-resubmission_SupplementaryFigure1.tif]

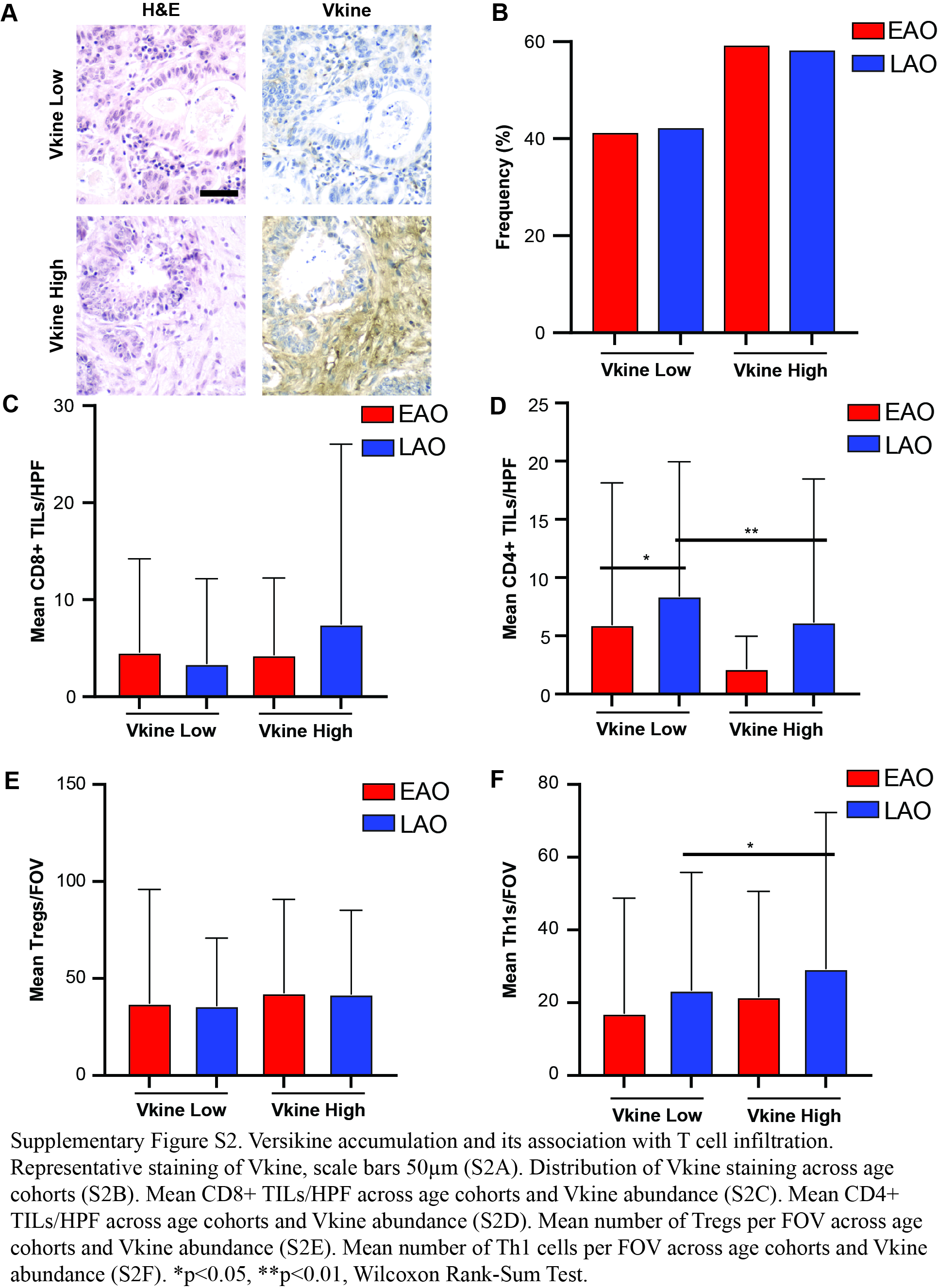

Supplement: goaf015_Supplementary_Data [file goaf015_supplementary_data.zip › Kraus_Johnson_EAO CRC Resubmission_SupplementaryFigure2wLegend.tif]
